# Crystal structure and DNA binding activity of a PadR family transcription regulator from hypervirulent *Clostridium difficile* R20291

**DOI:** 10.1186/s12866-016-0850-0

**Published:** 2016-10-04

**Authors:** Catherine E. Isom, Smita K. Menon, Leonard M. Thomas, Ann H. West, George B. Richter-Addo, Elizabeth A. Karr

**Affiliations:** 1 Price Family Foundation Institute of Structural Biology and Department of Microbiology and Plant Biology, University of Oklahoma, 770 Van Vleet Oval, Norman, OK 73019 USA; 2 Price Family Foundation Institute of Structural Biology and Department of Chemistry and Biochemistry, University of Oklahoma, 101 Stephenson Parkway, Norman, OK 73019 USA

**Keywords:** *Clostridium difficile*, Transcription regulation, Bacteria, Stress response, Structural biology, PadR

## Abstract

**Background:**

*Clostridium difficile* is a spore-forming obligate anaerobe that can remain viable for extended periods, even in the presence of antibiotics, which contributes to the persistence of this bacterium as a human pathogen during host-to-host transmission and in hospital environments. We examined the structure and function of a gene product with the locus tag CDR20291_0991 (*cd*PadR1) as part of our broader goal aimed at elucidating transcription regulatory mechanisms involved in virulence and antibiotic resistance of the recently emergent hypervirulent *C. difficile* strain R20291. *cd*PadR1 is genomically positioned near genes that are involved in stress response and virulence. In addition, it was previously reported that *cd*PadR1 and a homologue from the historical *C. difficile* strain 630 (CD630_1154) were differentially expressed when exposed to stressors, including antibiotics.

**Results:**

The crystal structure of *cd*PadR1 was determined to 1.9 Å resolution, which revealed that it belongs to the PadR-s2 subfamily of PadR transcriptional regulators. *cd*PadR1 binds its own promoter and other promoter regions from within the *C. difficile* R20291 genome. DNA binding experiments demonstrated that *cd*PadR1 binds a region comprised of inverted repeats and an AT-rich core with the predicted specific binding motif, GTACTAT(N_2_)ATTATA(N)AGTA, within its own promoter that is also present in 200 other regions in the *C. difficile* R20291 genome. Mutation of the highly conserved W in α4 of the effector binding/oligomerization domain, which is predicted to be involved in multi-drug recognition and dimerization in other PadR-s2 proteins, resulted in alterations of *cd*PadR1 binding to the predicted binding motif, potentially due to loss of higher order oligomerization.

**Conclusions:**

Our results indicate that *cd*PadR1 binds a region within its own promoter consisting of the binding motif GTACTAT(N_2_)ATTATA(N)AGTA and seems to associate non-specifically with longer DNA fragments *in vitro*, which may facilitate promoter and motif searching. This suggests that *cd*PadR1 acts as a transcriptional auto-regulator, binding specific sites within its own promoter, and is part of a broad gene regulatory network involved, in part, with environmental stress response, antibiotic resistance and virulence.

**Electronic supplementary material:**

The online version of this article (doi:10.1186/s12866-016-0850-0) contains supplementary material, which is available to authorized users.

## Background

Epidemiological trends indicate clinical acquisition of *Clostridium difficile* as the primary route of human infection by this bacterium [1]. The risk of *C. difficile* becoming a community-acquired infection is likely to increase without the development of better identification and more effective treatment [2]. The genome of *C. difficile* has been described as “highly dynamic” based on the prevalence of horizontal gene transfer [3]. The impact of a genome that readily changes in response to environmental stress could be a major indicator of *C. difficile* pathogenicity [3]. *C. difficile* produces spores that allow it to be viable for extended periods, even in the presence of antibiotics, which could explain the persistence of this human pathogen during host-to-host transmission and in the hospital environment [4]. Transcription factors orchestrate the regulation of survival, proliferation, virulence, and antibiotic resistance mechanisms of human pathogens. As part of our larger goal aimed at elucidating structure and function of transcription regulatory mechanisms involved in virulence and antibiotic resistance of human pathogens, we focused on protein targets from a hypervirulent strain of *C. difficile* (R20291). Herein, we present our results on a member of the PadR family of transcription regulators (product of CDR20291_0991) that we have named *cd*PadR1.

The first described PadR proteins are transcriptional repressors for genes encoding phenolic acid decarboxylase (*padC*) that de-repress *padC* when phenolic acids are present in toxic amounts [5]. The PadR transcription regulator from *Bacillus subtilis* is a prototypical PadR-family member protein that binds the *padC* promoter in the absence of phenolic acid *in vitro*; binding is lost when exposed to phenolic acids [6, 7]. Unlike the prototypical PadR, the PadR family transcription regulators AphA [8], LmrR [9], and *bc*PadR [10] from *Vibrio cholerae*, *Lactococcus lactis*, and *Bacillus cereus*, respectively, are involved in the regulation of virulence and antibiotic efflux mechanisms. The prototypical PadR and the PadR-like transcription regulator AphA are within a subfamily of PadR proteins (PadR-s1) which contain multiple α-helices in the C-terminal domain [10]. Another, less studied subfamily of PadR family proteins (PadR-s2), contains a single α-helix in the C-terminal effector binding/oligomerization domain [10]. The PadR-s2 proteins, which include the *bc*PadRs [10] and LmrR [11], have been structurally characterized and are involved in multiple drug recognition. The BC4206 gene product, *bc*PadR1, was upregulated 8.7-fold in the presence of enterocin treatment in *B. cereus* ATCC14572 when compared to an untreated control [12]. This PadR-like protein binds its own promoter and that of the gene BC4207, which encodes a membrane protein predicted to be involved in enterocin AS-48 resistance [12]. Binding of *bc*PadR1 to the predicted promoter region was not affected by the addition of AS-48 in vitro [10]. The PadR-like family protein of *L. lactis*, LmrR, binds the promoter region of an ABC-type multidrug transporter, LmrCD, and interacts with the compound Hoechst 33342 and the antibiotic daunomycin [9]. The crystal structure of apo-LmrR revealed a hydrophobic pore between α4 of the dimer mates [11]. Additional structures of LmrR bound to Hoechst 33342 and daunomycin, separately, demonstrated that this pore is integral to inhibitor interaction [11]. The conformational change instigated at α4 is predicted to interfere with DNA binding due to an increase in distance between α3 of the dimer mates [13]. This hydrophobic pore is not present in *bc*PadR structures determined to date.

The genome of hypervirulent *C. difficile* R20291 contains the protein coding sequence for three PadR-like family proteins (*cd*PadR1, CDR20291_1187, CDR20291_3068). The function of *cd*PadR1 is of interest due, in part, to its similarity to previously described *bc*PadRs and LmrR and the response of these transcription regulators to multiple inhibitors. Importantly, differential expression studies have linked *cd*PadR1 and a homologue from historical *C. difficile* strain 630 (CD630_1154) to regulatory networks that allow *C. difficile* to efficiently respond to environmental changes and, thus, survive within a host. This response is not necessarily due to direct interaction with stressors, but may be part of an overall regulatory cascade. Germination of *C. difficile* strain 630 endospores lead to the differential expression of 92 different transcriptional regulators, ~74 % of which were up-regulated as detected by microarray and validated by qRT-PCR [14]. Included in this list of differentially expressed transcription regulators is the *cd*PadR1 homologue CD630_1154, which was 2.3-fold up-regulated during germination [14]. This suggests that the expression of one or more of these proteins required to bring an endospore out of dormancy may be regulated by CD630_1154. Another study linked the differential expression of this *cd*PadR1 homologue to acid and alkali shock, oxygen exposure, and subinhibitory concentrations of metronidazole (Mtz) as detected by microarray analyses in *C. difficile* strain 630 [15].

Herein, we investigated the PadR-s2 protein from *C. difficile* strain R20291, *cd*PadR1. In this paper, we report the crystallization and X-ray crystal structure of *cd*PadR1 at 1.9 Å resolution. We also demonstrate *cd*PadR1 binding to its own gene promoter in a manner conducive to autoregulation. Additionally, we show that *cd*PadR1 binds the promoters of three additional regulatory signaling proteins and that a *cd*PadR1 binding motif is present upstream of 100 genes in *C. difficile* R20291.

## Methods

### Protein expression and purification

Residues 1-109 of *cd*PadR1 (locus tag CDR20291_0991) were amplified from gDNA using forward primer Pr3 –EAK (5′- TTCAGGGATCCATGCAGTTAAATAAAGAAGTGTTAAAAGG-3′) and reverse primer Pr4-EAK (5′-TTAAGCTGCAGTTAATCCACCTCTCCCAAAAATTG-3′) primers, each of which contained a 5 nucleotide overhang followed by restriction digestion sites for *Bam*HI (forward) or *Pst*I (reverse) for digestion and ligation into the expression vector. *cd*PadR1 was expressed in *Escherichia coli* Rosetta™ using the pQE80L (Qiagen) vector system modified to encode a *Strep* II™-tag on the N-terminus [16]. *cd*PadR1 was isolated by batch purification over Streptactin SuperFlow Plus resin (Qiagen). All buffers were prepared according to the manufacturers’ guidelines. Cell lysis, column equilibration, and wash buffer contained 50 mM NaH_2_PO_4_ and 300 mM NaCl (pH 8.0 using NaOH). Elution buffer contained 50 mM NaH_2_PO_4_, 300 mM NaCl, and 2.5 mM d-desthiobiotin (pH 8.0 using NaOH). Subsequent purification of the *cd*PadR1 dimer was accomplished by size exclusion chromatography in buffer containing 20 mM Tris (pH 8.0 with NaOH) and 150 mM NaCl, using a Superdex 200 Increase 10/300 GL column connected to an ÄKTA Pure 25 (GE Healthcare). Fractions corresponding to a dimer were concentrated using Amicon® concentration units (Millipore) primed with glycerol and buffer exchanged into 10 mM Tris (pH 8.0) and 100 mM KCl. The molecular weight (MW) was determined by coupling SEC with multi-angle light scattering (MALS) and outputs were analyzed by the ASTRA software (Wyatt Technology).

### Crystallization of *cd*PadR1

Crystals were initially obtained by vapor diffusion using a MCSG Crystallization Suite (Microlytic) (3 M NaCl and 0.1 M HEPES pH 7.5) with a final protein concentration of 1.5 mg mL^-1^. Crystal growth was optimized at room temperature by hanging drop vapor diffusion with the drops containing 3 μL protein solution (4 mg mL^-1^ *cd*PadR1 in 100 mM KCl, 10 mM Tris pH 8.0) and 1 μL reservoir solution (3.1 M NaCl, 100 mM HEPES [pH 7.5]). Crystals were transferred into drops containing an equal volume of 2X reservoir solution and 40 % glycerol for cryoprotection. Crystals in cryosolution were incubated over original well solution for 5 min before freezing in a liquid nitrogen gas stream for cryogenic data collection.

### Data collection and structure determination

X-ray diffraction data were collected using a MARmosaic325 CCD detector at the Stanford Synchrotron Radiation Lightsource (SLAC National Accelerator Laboratory) on beam-line BL14-1. The data were processed with XDS and XSCALE [17]. The XDS output files were converted to .mtz format using CCP4 [18]. The structure of *Clostridium thermocellum* PadR-like family protein (*Ct*PadR, PDB ID 1XMA) was used as the starting model for molecular replacement using Phaser-MR [19]. The individual coordinates of the preliminary model were generated in AUTOBUILD [20], were refined and rebuilt using the model in COOT [21] and any positions with strong densities outside of the model were accounted for. Structure alignments were performed in COOT and all structure/alignment figures prepared using PyMOL [22]. Residues 1–9, 41, and 107–109 were not modeled due to the absence of electron density. Coordinates have been deposited with the Protein Data Bank (www.rcsb.org) with PDB ID 5DYM. Data collection and refinement statistics are shown in Table 1.

**Table 1:** Data collection and refinement statistics

Data collection statistics (SSRL BL14-1)	
Wavelength (Å)	1.2
Space group	P 4_1_ 2_1_ 2
Unit cell dimensions	
*a, b, c* (Å)	92.4, 92.4, 40.4
α, β, γ (°)	90
Resolution (Å)	34.4 – 1.9 (2.0 – 1.9)^a^
*R*_*merge*_^*b*^	0.019 (0.38)
*I/σ[I]*	20.3 (2.2)
Completeness (%)	99 (95)
Redundancy	2.0 (2.0)
Wilson *B* (Å)	33.1
Refinement statistics	
Resolution range (Å)	34.4 – 1.9 (2.0 – 1.9)
No. of reflections	
Total	28484 (2641)
Unique	14302 (1332)
*R*_*work*_^c^ (%)	0.21 (0.29)
*R*_*free*_^d^ (%)	0.23 (0.34)
No. of atoms	
Total (non-H)	842
Water molecules	34
Mean overall B factors	
Protein	49.3
Solvent	43.3
RMSDs	
Bond length (Å)	0.008
Bond angles (°)	1.03
Ramachandran plot	
% most favored residues	96
% outliers	0
PDB code	5DYM

^a^Statistics for data in the highest resolution shell are given in parentheses

^b^ *R*_*merge*_ = (Σ*I*_obs_ - *I*_avg_)/Σ*I*_avg_

^c^ *R*_*work*_ = (Σ*F*_obs_ - *F*_calc_)/Σ*F*_obs_

^d^ *R*_*free*_ was calculated using the test set obtained by randomly selecting 10 % of the data

### Construction of *cd*PadR1^W94A^

The tryptophan 94 (W94) codon (TGG) of *cd*PadR1 was converted to alanine (GCG) by overlapping PCR [23]. The sequence of forward and reverse primers used to generate the alanine codon substitutions in *cd*PadR1 were 5′-GAAACAAGAAGCGAGATTTATTAAAAAG-3′ and 5′-CTTTTTAATAAATCTCGCTTCTTGTTTC-3′, respectively. The resulting plasmid was confirmed by sequencing, and the resulting protein variant was overexpressed and purified in the same manner as performed for the native *cd*PadR1.

### Electrophoretic Mobility Shift Assay (EMSA)

Double stranded DNA fragments for EMSA were generated by suspending custom complementary ssDNA (LifeTechnologies) in annealing buffer (10 mM Tris [pH 8.0] and 50 mM NaCl) and heating to 95 °C for 5 min followed by slowly cooling to room temperature. DNA was quantified with the Quant-IT™ Broad Range DNA assay and a Qubit® fluorimeter (Invitrogen). Template dilutions for EMSA stock solutions were dependent on the size of the DNA fragment and ranged from 0.5 μM (100 bp fragment) to 2.5 μM (20 bp fragment). Binding reactions were performed at room temperature. Each reaction mixture contained 20 mM Tris pH 8.0, 120 mM KCl, 12.5 % glycerol, 10 mM MgCl_2_, 5 mM DTT, and 125 μg mL^-1^ heparin. Heparin concentration was increased to 400 μg ml^-1^ for competition studies. A 1:10 dilution of DNA stock was added to all reactions and a *cd*PadR1 concentration 2.5-40-fold greater than that of final DNA concentration was added to start the binding reaction. A protein-free control was also included. EMSAs were performed in 8 % polyacrylamide gels and TB running buffer (89 mM Tris base and 89 mM boric acid) at 200 V and 20–100 mA with run time ranging from 20 min (20 bp fragments) to 30 min (100 bp fragments). Gels were stained with SYBR® Gold Stain (Invitrogen). Image coloration was inverted for ease of viewing. A list of oligonucleotides examined, including location on the genome, sequences, and GC content (%) can be found in Additional file 6: Table S1.

### Motif Analysis

GLAM2 was utilized to find a representative *cd*PadR1 motif [24]. The sequence surrounding Boxes 1 & 2 (5′-GTACTATACATTATAGAGTAGTAG-3′) and Boxes 3 & 4 (5′-AGAGTACTATGTATTATTATAGTAAAT-3′) were used as input sequences for the GLAM2 analysis. The GLAM2 search was done using the default parameters and allowed the motif sites to be on either the plus or minus strand. The direct GLAM2 output was used as the input for GLAM2 Scan using the *C. difficile* R20291 genome. Motifs were allowable on either the minus or plus strand of the genome and 200 alignments were allowed. The identified motifs were then mapped onto the *C. difficile* R20291 genome sequence in Geneious v8 [25]. The motifs were then manually curated to determine whether they were located within an open reading frame, an intergenic promoter region or between convergent genes.

## Results and discussion

### Crystal structure of recombinant *cd*PadR1

**Fig. 1:**
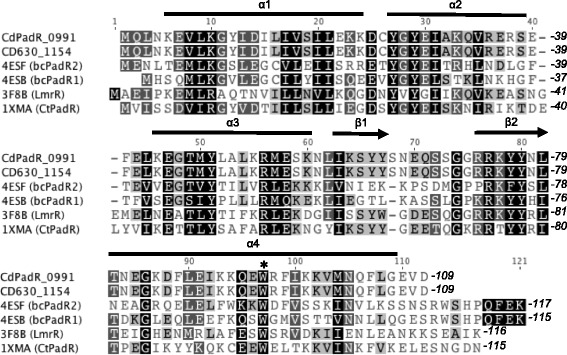
Amino acid sequence alignment of *cd*PadR1 from *Clostridium difficile* R20291 (CDR20291_0991) and 630 (CD630_1154) with structural homologues listed by accession number as follows: 4ESB (*bc*PadR2) from *Bacillus cereus* ATCC 10987 [10], 4ESF (*bc*PadR1) from *B. cereus* ATCC 14579 [10], 3F8B (LmrR) from *Lactococcus lactis* MG1363 [11], and 1XMA (*Ct*PadR) from *Clostridium thermocellum* (unpublished). *cd*PadR1 and CD630_1154 share 100 % amino acid sequence identity. Conserved residues are shaded black. Alpha helices are indicated by black bars and β-sheets are indicated with black arrows. The highly conserved W residue is demarcated with a black asterisk (*)

*cd*PadR1 shares 100 % amino acid sequence identity with the PadR-like transcription regulator, CD630_1154, in the historical *C. difficile* strain 630 (Fig. 1), both of which were differentially expressed under conditions of environmental stress [15]. *cd*PadR1 crystallized in space group P4_1_2_1_2 and, following X-ray data collection, the structure was solved by molecular replacement using the PadR family protein from *C. thermocellum* (*Ct*PadR) as a search model (PDB ID 1XMA). *Ct*PadR and *cd*PadR1 share 42 % amino acid sequence identity (Fig. 1) and, based on 3D prediction programs [26, 27], were expected to have high structural similarity (RMSD = 1.7 Å). The model was refined to a final crystallographic R-factor of 21.0 % (R_free_ = 23.0 %) (Table 1).

**Fig. 2:**
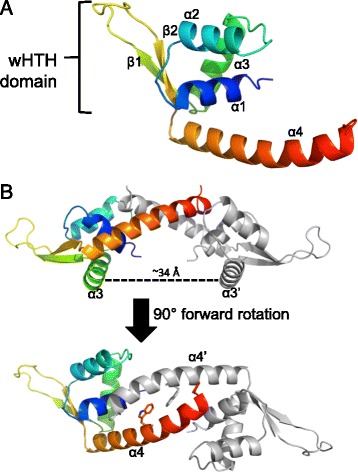
Overall structure of *cd*PadR1. **a** Ribbon representation of *cd*PadR1 monomer with a rainbow color gradient from the N-terminus (blue) to the C-terminus (red). Alpha helices and β-sheets are labeled numerically. The winged helix-turn-helix (wHTH) DNA binding domain is indicated. **b** The *cd*PadR1 structure is shown perpendicular to the two-fold axis of symmetry with the DNA recognition helices indicated (α3/α3′). The dimer mate is shown in gray. Distance between α3/α3′ was estimated in PyMOL [22]. Another view is shown after a 90° forward rotation which results in a view along the two-fold axis facing α4/α4′ with the conserved W residue (sticks)

One molecule of *cd*PadR1 was present in the asymmetric unit and consists of an N-terminal winged helix-turn-helix (wHTH) domain (residues 6–80) and a single α-helical C-terminal domain (residues 81–106) (Fig. 2a). This small C-terminal domain places *cd*PadR1 in the PadR-s2 subfamily of PadR transcriptional regulators described previously [10]. *cd*PadR1 forms a dimer with a 2-fold crystallographic axis of symmetry (Fig. 2b), similar to the *bc*PadRs (PDB IDs 4ESB and 4ESF) and LmrR (PDB ID 3F8B), both of which are PadR-s2 family proteins. The dimeric state of *cd*PadR1 is retained in solution as determined by size exclusion chromatography (Additional file 1: Figure S1). The recognition helices (α3/α3′) are positioned ~34 Å apart (Fig. 2b) consistent with symmetrical binding to two “half-sites” approximately 10 bp in length [28]. Dimerization of *cd*PadR1 buries approximately 1100 Å^2^ solvent-accessible surface area (16 %) of the approximately 7000 Å^2^ total solvent-accessible area per subunit [29]. Residues on helices α1, α2, and α4 that interact to form the *cd*PadR1 dimer interface are conserved across structural homologues (Fig. 1). The RMSD values for the *cd*PadR1 structural homologues *bc*PadR1, *bc*PadR2, apo-LmrR, LmrR-H33342, and LmrR-daunomycin are 1.6 Å, 1.6 Å, 2.1 Å, 2.9 Å, and 3.3 Å, respectively [27].

**Fig. 3:**
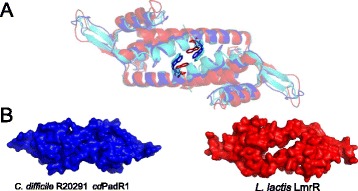
Differences between *cd*PadR1, *bc*PadR1 (PDB ID 4ESB), and apo-LmrR (PDB ID 3F8B). **a** Superposition of *cd*PadR1 (blue), *bc*PadR1 (cyan), and LmrR (red) in cartoon representation with the conserved W indicated (sticks). **b** Surface representations of *cd*PadR1 and apo-LmrR along the two-fold axis facing α4/α4′ (orientation same as Fig. 3c), which highlights the hydrophobic pore in LmrR and the closed dimeric interface of *cd*PadR1

The primary helices involved in dimerization are α1 and α4. The amino acid sequence pairwise identities between α1 of *cd*PadR1 and *bc*PadR1, *bc*PadR2, and LmrR are 26 %, 35 %, and 21 %, respectively. The amino acid sequence pairwise identity between α4 of *cd*PadR1 and *bc*PadRs (22.2 and 33.3 % for *bc*PadR1 and *bc*PadR2, respectively) is higher than the identity between α4 of *cd*PadR1 and LmrR (15 %). Helix α4 and α4′of *cd*PadR1 bend toward each other (Fig. 2b) and interact via a coiled-coil, whereas α4 and α4′ of LmrR do not display a significant bending towards each other at the C-terminus (Fig. 3a, red). In addition, LmrR contains fewer residues involved in dimerization at the C-terminus of the helix than *cd*PadR1 and *bc*PadRs. *cd*PadR1, like *bc*PadRs and *ct*PadR, has a closed dimeric interface, unlike the hydrophobic pore wherein aromatic drug-interaction occurs in LmrR (Fig. 3b). The known structural homologues of *cd*PadR1 contain a conserved W located within residues 91–96 in the α4 helix region that is predicted to be involved in both dimerization and drug binding [10, 11]. The distance between the conserved W residues in the α4 helix dimer mates for *cd*PadR1, *bc*PadR1 (4ESB), apo-LmrR (3F8B), LmrR-H33342 (3F8C), and LmrR-daunomycin (3F8F) was measured using Chimera [30]. Distances were determined from the centroids of the phenol rings (P-P), indole rings (I-I), and indole-to-phenol (I-P) of the conserved α4 W residues. The P-P, I-I, and I-P distances between *cd*PadR1 W94 and W94′ are 5.4 Å, 9.2 Å, and 7.4 Å, respectively. These distances are similar to those of *bc*PadR1 (P-P = 5.6 Å, I-I = 9.1 Å, and I-P = 7.4 Å). The P-P distance is ~2 Å greater for the apo-LmrR (P-P = 6.9 Å), LmrR-H33342 (P-P = 7.2 Å), and LmrR-daunomycin (P-P = 7.4 Å) structures when compared to the distance between phenol centroids in *cd*PadR1. The increased distance between α4 and α4′of LmrR allows for aromatic inhibitor interaction via π-stacking between the W96 and W96′ residues [11]. The lack of a drug-binding pocket in *cd*PadR1 suggests that any differential expression of the CD630 homologue (CD630_1154) during Mtz exposure would, most likely, be due to a regulatory cascade effect rather than direct interaction of *cd*PadR1 with Mtz. It was suggested that changes in the orientation of α4 and α4′in a drug-bound state effects the position of the DNA recognition helices, rotating them away from each other [11]. This, presumably, would cause a change in DNA-binding. Previous work revealed that LmrR binds two sites within the *lmrCD* promoter, one region containing the predicted -10 and -35 sites and the other containing the inverted repeats ATGT/ACAT separated by 10 nucleotides and that this is consistent with a “conserved” binding motif among other PadR-like regulators with an eight nucleotide linker between the inverted repeats ATGT/ACAT [9, 31]. The recognition helices (α3/ α3’) are positioned ~34 Å apart in *cd*PadR1 (Fig. 2b), which is consistent with symmetrical binding to two “half-sites” comprised of inverted repeats ~10 bp apart; it is important to note that this distance does not account for DNA secondary structure. DNA binding behavior was explored for *cd*PadR1 to determine if it functions similarly to previously studied PadR family transcription regulators and to begin elucidating the regulatory networks of *cd*PadR1 in hypervirulent *C. difficile* in vitro.

### *cd*PadR1 binding to its own promoter

**Fig. 4:**
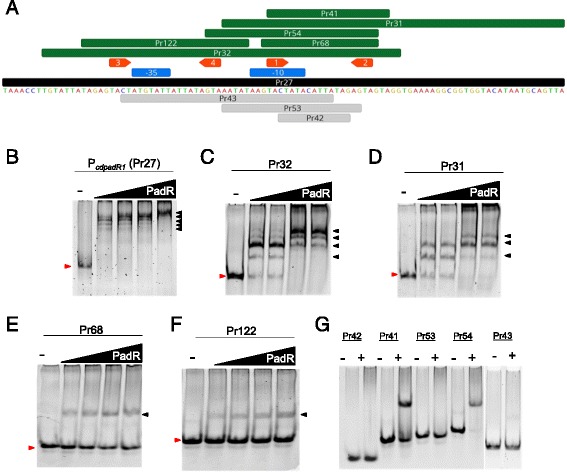
EMSAs of *cd*PadR1 binding the *cdpadR1* promoter (P_*cdpadR1*_) and smaller regions containing predicted binding boxes. **a** P_*cdpadR1*_ fragments that were bound by *cd*PadR1 are illustrated in green above and those that were not bound are illustrated in gray below the P_*cdpadR1*_ sequence. The predicted -10 and -35 sites are indicated in blue boxes above the sequence. **b** Final P_*cdpadR1*_ (Pr27, 100 bp) concentration in the reaction was 0.05 μM. *cd*PadR1 was 5, 10, 20, and 40-fold excess over DNA. **c**-**d** Final Pr32 (64 bp) and Pr31 (61 bp) concentration in the reactions were 0.1 μM. *cd*PadR1 was 5, 10, 20, and 40-fold excess over DNA. **e**-**f** The 21 bp P_*cdpadR1*_ fragment (Pr68) contains predicted binding boxes 1 and 2 (orange arrows) separated by 11 bp and contains a 1 bp overhang on both the 5′ and 3′ ends. The 30 bp P_*cdpadR1*_ fragment (Pr122) contains predicted binding boxes 3 and 4 (orange arrows) and contains 5 bp overhangs on both the 5′ and 3′ ends. Final Pr68 (21 bp) and Pr122 (30 bp) concentration in the reactions were 0.25 μM. *cd*PadR1 was 2, 4, 6, and 8-fold excess over DNA. **g** Final dsDNA concentration varied depending on the size of the fragment; the + lane contains 10-fold excess *cd*PadR1 over DNA. For EMSA gels B-F the minus (-) lane contains DNA and no *cd*PadR1. Shifted DNA-protein complexes are annotated with a black arrow and unbound DNA migration is marked with a red arrow

A 100 bp region upstream of *cdpadR1* (P_*cdpadR1*_/Pr27) was used in EMSA assays to determine if *cd*PadR1 binds its own promoter (Fig. 4a). The presence of five bands with differing mobility indicated that protein-DNA complexes of varying stoichiometry were produced. This may be the result of multiple binding sites and/or higher order oligomerization upon DNA binding (Fig. 4b). Increasing the concentration of *cd*PadR1 in the reaction resulted in a variation of the migration pattern until an observed saturation point at the slowest mobility compared to other bands was achieved (40-fold *cd*PadR over DNA or 4 μM *cd*PadR1, Fig. 4b, far right). *cd*PadR1 binding to P_*cdpadR1*_ (Pr27) is consistent with auto-regulation of its own expression.

To further define the binding sites for *cd*PadR1 binding to P_*cdpadR1*_, Palinsight was used to identify inverted repeats within Pr27 characteristic of those bound by transcriptional regulators containing a HTH motif [32–34]. Two sets of inverted repeats (Box 1/2 and 3/4) were identified with a TACT(N_11-12_)AGTA sequence motif (Fig. 4a). A series of smaller dsDNA fragments within the 100 bp P_*cdpadR1*_ were designed to test the role of these inverted repeats in *cd*PadR1 binding to P_*cdpadR1*_ (Fig. 4a). A 64 bp fragment containing both sets of inverted repeats (Pr32) showed four shifts of varying stoichiometry similar to that seen for Pr27 (Fig. 4c). However, full saturation, as seen for Pr27, was not achieved suggesting that additional space on the DNA for higher order oligomerization is needed to see complete shifting to one higher molecular weight complex. When *cd*PadR1 bound a 61 bp fragment that contained only one set of inverted repeats (Pr31) three shifted complexes were observed (Fig. 4d). This is consistent with the loss of a full binding site and additional space on the DNA for higher order oligomerization as noted for Pr31.

We further narrowed *cd*PadR1 binding to two small regions of P_*cdpadR1*_ (Pr68 and Pr122) each containing one set of inverted repeats TACT(N_11-12_)AGTA (Fig. 4a). *cd*PadR1 bound the 21 (Pr68) and 30 bp (Pr122) regions of P_*cdpadR1*_ with a single stoichiometry as visualized using EMSA (Fig. 4e and f, respectively). Additionally, a variety of dsDNA fragments representing various sub-regions of the original 100 bp P_*cdpadR1*_ (Pr27) were examined and, unless the fragment contained the predicted inverted repeats TACT(N_11-12_)AGTA, no binding was observed (Fig. 4g). It was noted that the N_11-12_ spacer region within the inverted repeats was AT rich. To determine whether the AT richness contributes to localized bending of the DNA that facilitates binding we replaced the TTATA in Pr68 with a GCCTG sequence (Pr101). Indeed, significant binding of *cd*PadR1 to Pr101 was not observed (Additional file 2: Figure S2) suggesting that the AT-rich spacer is important for binding. It should be noted that a fragment containing the AT rich portion but lacking the intact TACT/AGTA (Pr42) was not bound by *cd*PadR1 (Fig. 4a and g). This indicates that the AT rich sequence is not the direct binding site for *cd*PadR1. Additionally, varying the length of the spacer between the TACT/AGTA inverted repeat in Pr68 did not interfere with binding (Additional file 2: Figure S2) suggesting that flexibility of the DNA region between the inverted repeat rather than the length is more important for *cd*PadR1 binding.

To summarize, *cd*PadR1 binding to P_*cdpadR1*_ is dependent upon a TACT/AGTA inverted repeat sequence. Two such sequences are present in the 100 bp P_*cdpadR1*_investigated in this study. These two inverted repeats are responsible for two sequence-specific interactions between *cd*PadR1 and its promoter that can account for two shifted complexes. Additional shifted complexes may be the result of higher order oligomerization of *cd*PadR1 once bound to DNA or a decrease in the constraints on sequence specificity. Although constraints on the spacing between the TACT/AGTA inverted repeats do not appear to be tight, there does appear to be a requirement for AT richness within the spacer. The placement of the inverted repeat within P_*cdpadR1*_ is consistent with auto regulation. *cd*PadR1 and the *cd*PadR1 homologue CD630_1154 both contain TACT/AGTA with an 11 nucleotide spacer 25 bp upstream of the open reading frame (ORF). Additionally, both promoter regions contain TACT(N_12_)AGTA 52 bp upstream of their respective ORFs and overlapping the predicted -35/-10 promoter region, which suggests a similar binding function for each of these genes to their respective promoters.

### *cd*PadR1 binds other gene promoters with the *cd*PadR1 motif

**Fig. 5:**
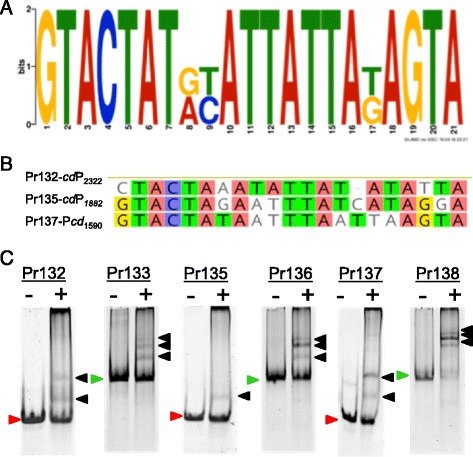
Example analysis of predicted *cd*PadR1 binding motif (*cd*PadR1 motif) as verified by EMSA. **a** Motif identified by GLAM2 analysis [24] of small dsDNA fragments from within the *cd*PadR1 promoter (Pr68 and PR122). **b** *cd*PadR1 motifs present in three exemplar promoters compared to the *cd*PadR1 motif. Nucleotides that are in agreement with the reference *cd*PadR1 motif are highlighted. Pr132 and Pr133 are upstream of CDR20291_2322 (IclR family transcription regulator CDS); Pr135 and Pr136 are upstream of CDR20291_1882 (two-component system response regulator CDS); Pr137 and Pr138 are upstream of CDR20291_1590 (ArsR family transcriptional regulator CDS). **b** EMSAs of *cd*PadR1 binding 30 bp (green arrows) and 100 bp (red arrows) promoter regions that contain *cd*PadR1 motif. DNA only (-) reactions contained 100 nM either 30 or 100 bp dsDNA from promoters listed above. Reactions containing dsDNA and 4 μM *cd*PadR1 are noted with a plus (+) sign. Final DNA concentration in both reactions was 100 nM. Bound complexes are indicated with black arrows

The dsDNA fragments containing TACT(N_11-12_)AGTA from *P*_*cdPadR1*_ were analyzed for conserved binding motif using GLAM2 [24]. GLAM2 was advantageous over MEME because it allows for spacing/gaps in motif prediction since spacing between the inverted repeats was not critical for binding. The best motif was 21 bp in length with the sequence GTACTAT(N_2_)ATTATA(N)AGTA and was designated *cd*PadR1 motif (Fig. 5a). GLAM2Scan results indicated the presence of 200 potential motif matches in the *C. difficile* strain R20291 genome with scores ranging from 13.6–18.7, not including the P_*cdpadR1*_ sequences used for analysis (Additional file 3: Table S2). Approximately half of these motifs are either situated between two convergent genes or are located within open reading frames (ORFs). Of those that are located upstream of genes, approximately 6 % are upstream of other transcription regulators and other regulatory proteins, such as two-component response regulators, while another ~7 % are upstream of genes involved in transport/efflux and sporulation. The genes predicted to be involved in transport/efflux are the ABC transporter ATP-binding proteins CDR20291_0159, _0296, _0551, _0553, and _3203 (Additional file 4: Table S3). Two genes predicted to be involved in sporulation also contain the *cd*PadR1 binding motif upstream of the transcription start site, a spore maturation protein (CDR20291_3377) and a spore coat assembly protein (CDR20291_0316) (Additional file 4: Table S3). Over 50 % of the predicted binding motifs were indicated to be either upstream genes of “hypothetical proteins”, within open reading frame, or between convergent genes. Exemplar promoters from this list were selected for analysis using EMSA to determine binding of *cd*PadR1 to these promoter fragments in vitro (Fig. 5b and c). A 30 bp and 100 bp dsDNA fragment was selected for each promoter region and contained at least one predicted *cd*PadR1 motif (Fig. 5b and c). Pr132 and Pr133 contain the *cd*PadR1 motif located 45 base pairs upstream of CDR20291_2322 (IclR family transcription regulator CDS) (*cd*P_*2322*_). Pr135 and Pr136 contain the *cd*PadR1 motif located 116 base pairs upstream of CDR20291_1882 (two-component system response regulator CDS) (*cd*P_*1882*_). Pr137 and Pr138 contain the *cd*PadR1 motif located 25 base pairs upstream of CDR20291_1590 (ArsR family transcriptional regulator CDS) (*cd*P_*1590*_). *cd*PadR1 bound all of the selected promoters *in vitro* (Fig. 5c). The 30 bp promoters (Pr132, Pr135, Pr137) yielded two discrete bands. However, this phenomenon has also been observed on occasion for the short dsDNA fragment containing one set of inverted repeats from P_*cdpadR1*_ (Pr68, Additional file 5: Figure S3) and is not likely to represent multiple binding events to a small dsDNA fragment [35]. This binding pattern may be attributable to the presence of small amounts of ssDNA, portions of the dsDNA with secondary structure, or conformational changes in the DNA upon binding in a small subset of the complexes which are more pronounced in shorter dsDNA fragments [35].

Gene regulatory networks play an integral role in the physiology of microorganisms and their response to ever changing environments [36, 37]. The binding of *cd*PadR1 to the promoters of genes encoding transcription regulators and a DNA-binding response regulator, part of a two-component signal transduction system, suggests it may play a role in a gene regulatory network in *C. difficile*. The *cd*PadR1 motif overlaps the predicted -10 region of *cd*P_*1590*_ and *cd*P_*2322*_ [38]. This positioning of a regulatory binding site overlapping the -10 region is consistent with repression via abrogation of the Sigma factor. In *cd*P_*1882*_, the *cd*PadR1 motif is located approximately 30 bp upstream of the predicted -35 region [38]. Positioning of a regulatory binding site upstream of the -35/-10 core promoter elements is typically consistent with a role in activation of the promoter [39]. While additional studies are necessary to determine the biological role of *cd*PadR1 in activation or repression of these promoters, it is notable that *cd*PadR1 is able to bind these promoters and likely participates in a regulatory cascade in response to undetermined stimuli.

### *cd*PadR1 binds other promoter regions

**Fig. 6:**
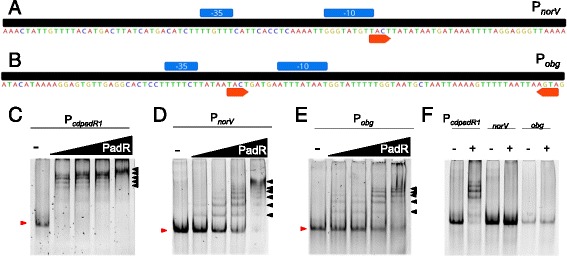
EMSAs of *cd*PadR1 binding the *cdpadR1* promoter (P_*cdpadR1*_), **a** *norV* promoter (P_*norV*_), and **b** *obg* promoter (P_*obg*_). The predicted -10 and -35 sites are indicated in blue boxes above the sequence. Orange arrows indicate the inverted repeats TACT/AGTA. **c**-**e** Final P_*cdpadR1,*_ P_*norV*_, and P_*obg*_ (100 bp) concentration in the reaction was 0.1 μM. *cd*PadR1 was 5, 10, 20, and 40-fold excess over dsDNA. The minus (-) lane contains dsDNA and no *cd*PadR1. Shifted DNA-protein complexes are annotated with a black arrow and unbound dsDNA migration is marked with a red arrow. **f** For this EMSA, the final heparin concentration was 4-fold of the standard EMSA concentration used throughout this research. The + lane contains 40-fold excess *cd*PadR1 over dsDNA

Additional promoters from the *cdpadR1* genomic neighborhood were chosen to test for *cd*PadR1 binding based on gene expression studies. A promoter for a nitric oxide reductase (*norV*, CDR20291_0994) and a Spo0B-associated GTP-binding protein were selected. Nitric oxide reductase has been linked to pathogenesis in other microorganisms [40] and was 2-fold down regulated, along with *cd*PadR1 when compared to the historical *C. difficile* strain 630 [41]. Another representative promoter for EMSA study from within *cd*PadR1 genome neighborhood is upstream of a gene encoding a Spo0B-associated GTP-binding protein (*obg*, CDR20291_1001) whose homologue was 2-fold down regulated following pig loop infection with the historical strain *C. difficile* 630 [42]. *cd*PadR1 bound P_*norV*_ and P_*obg*_ in vitro (Fig. 6d and e, respectively). The migration patterns for P_*norV*_ and P_*obg*_ differ from that of P_*cdpadR1*_ (Fig. 6c). For all promoters examined, slower migrating complexes appeared at increasing protein concentrations, which suggests that *cd*PadR1 binds to multiple sites in the upstream region of the gene. However, the complexes formed when *cd*PadR1 is incubated with promoters other than its own are smaller and it appears that a level of saturation, wherein only one large complex is formed, is not reached as it is for P_*cdpadR1*_. It is well understood that transcription regulators bind a relatively limited set of DNA sequences [43], a concept that we explored for *cd*PadR1 and P _*cdPadR1*_ (Fig. 4), as well as a predicted binding motif (*cd*PadR1 motif, Fig. 5). Both P_*norV*_ and P_*obg*_ have only one half of the inverted repeat within the *cd*PadR1 motif (Fig. 6a and b). However, it is unclear whether only one half-site is sufficient to initiate binding to these promoters or if perhaps the binding is non-specific and related to local DNA structure or AT content. Therefore, we examined binding specificity using increased amounts of heparin as a competitor for *cd*PadR1 binding (Fig. 6f). When a 4-fold higher concentration of heparin was present in the binding reaction of *cd*PadR1 to P_*norV*_ or P_*obg*_ a shifted complex was no longer detected at 40-fold protein over dsDNA (Fig. 6f). Under the same conditions, *cd*PadR1 still bound its own promoter, though the larger complexes were no longer detected. That *cd*PadR1, a small HTH DNA binding protein, would bind other 100 bp predicted promoter regions non-specifically could be explained using the theoretical model termed one-dimensional diffusion, or “sliding”. During one-dimensional diffusion, the transcriptional regulator searches for specific binding sites along the DNA remaining in contact with the DNA due to non-specific interactions [44–46]. It is, therefore, likely that a more specific level of binding requires the full *cd*PadR1 motif. So, while *cd*PadR1 does, in fact, bind P_*norV*_ and other 100 bp AT-rich promoters in vitro (Additional file 6: Table S1), no conclusions can be made regarding the regulation of this or any other promoters tested based on EMSA alone. Coupled with the recent expression studies, however, in vitro binding assays suggest that further study into the regulation of expression of these genes, especially *norV*, is warranted.

### Role of the conserved W residue in *cd*PadR1 DNA binding

**Fig. 7:**
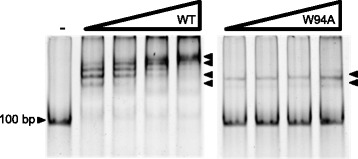
EMSA of *cd*PadR1^WT^ and *cd*PadR1^W94A^ binding P_*cdpadR1*_. Protein-free controls are indicated with a minus sign (-). 100 bp P_*cdpadR1*_DNA (0.05 μM) was used in EMSA to determine binding of *cd*PadR1 to its own promoter at increasing concentrations (0.25, 0.50, 1.0, and 2.0 μM) of protein

It was suggested previously that the conformational changes elicited by drug binding between α4/α4′ could affect DNA binding and that a conserved tryptophan (W) in α4 was directly involved in drug binding; an indirect role of this W residue was indicated in DNA binding [11]. We examined the effect of this conserved W at residue 94 (W94) in *cd*PadR1 on DNA binding in vitro (Fig. 7). When W94 is replaced with an alanine (*cd*PadR1^W94A^), the majority of binding along with the higher order complexes observed for *cd*PadR1^WT^ binding to P_*cdpadR1*_ are lost (Fig. 7). Dimerization was not effected as detected by size exclusion chromatography coupled with multi-angle light scattering detection (SEC-MALS, Additional file 1: Figure S1). These results suggest that, while the conserved W does not affect dimerization, it does inhibit DNA binding in vitro in a way that is not entirely clear while further supporting a role of the conserved W in DNA binding. The suggested mechanism by Madoori et al wherein the DNA binding helices of LmrR putatively rotate away from each other when the effector-binding/oligomerizatoin domain is perturbed at the conserved W residue is further supported as the mechanism of lowered DNA binding affinity by the results presented here.

## Conclusion

We have determined the 1.9 Å resolution crystal structure of *cd*PadR1, which revealed that it is in the PadR-s2 subfamily of PadR transcriptional regulators with other structurally and functionally characterized PadR-like regulators from *B. cereus* (*bc*PadR1 and *bc*PadR2) and *L. lactis* (LmrR). In vitro protein-DNA binding experiments demonstrate that *cd*PadR1 binds a region comprised of the inverted repeats TACT/AGTA and an AT-rich core, GTACTAT(N_2_)ATTATA(N)AGTA, within its own promoter. These predicted binding sites are present in the *cd*PadR1 homologue CD630_1154, suggesting that these transcription regulators are functional homologues as well. *cd*PadR1 appears to be part of a hierarchical gene regulatory network in *C. difficile*. Furthermore, *cd*PadR1 non-specifically associates with longer DNA fragments that may facilitate promoter and motif searching. Mutation of the highly conserved W in the α4 helical region, which is predicted to be involved in multi-drug recognition and dimerization in LmrR, resulted in alterations of *cd*PadR1 binding to the predicted binding motif, potentially due to tighter constraints on spacing of the inverted repeats as well as a loss of higher order oligomerization. Complementary in vivo studies of *cd*PadR1 will allow for a better understanding of its regulatory network.

## Additional files

Additional file 1: Figure S1. (A) Chromatogram from size exclusion chromatography (SEC) run performed on a Superdex 200 Increase 10/300 GL column connected to an ÄKTA Pure 25 (GE Healthcare). The black line represents the calibration standard mix with molecular weights of standards labeled. The blue and red lines indicate the elution profile for *cd*PadR1^WT^ and *cd*PadR1^W94A^, respectively (13.5 kDa monomer size for both). (B) Molar mass versus elution time of *cd*PadR1^WT^ and *cd*PadR1^W94A^ from SEC (as described) coupled with multi-angle light scattering (MALS) detection. Red lines indicate MALS signal (LS) and green lines indicate UV detection. *cd*PadR1^WT^ and *cd*PadR1^W94A^ both dimers with molecular weights (MW) of approximately 30 and 27 kDa, respectively (monomeric *cd*PadR1 MW is 13.5 kDa). (PPTX 222 kb)
Additional file 2: Figure S2. EMSA of *cd*PadR1 binding different fragments of P_*cdpadR1*_, that contain the inverted repeats TACT/AGTA with 4 bp overhang on the 5′ and 3′ end of inverted repeats. Each dsDNA contains a different number of nucleotides between TACT/AGTA (addition of a central alanine). EMSAs were conducted as described for 100 bp and smaller dsDNA fragments in the materials and methods. A complete list of nucleotides tested is available in the table below the EMSA gels. Inverted repeats are underlined. For Pr101, the AT rich region that was mutated is indicated in bold. The - lane contains DNA only and the + lane contains 10-fold *cd*PadR1 in excess over DNA. (PPTX 256 kb)
Additional file 3: Table S2. List of GLAM2Scan [24] results for scan of the *Clostridium difficile* R20291 genome for *cd*PadR1 motif (Fig. 5a). The.xlsx file includes the locus ID for the gene(s) nearest the motif (column A); the gene annotation, if applicable, or the indication that this motif is within an open reading frame (ORF) or between two convergent genes (column B); the genomic placement (start, column C and end, column D); indication of whether the motif is on the leading (+) or lagging (-) strand (column E); the motif sequence generated by GLAM2Scan (column F); and the score generated by GLAM2Scan that indicates how well the sequence fits the *cd*PadR1 motif (column G). (XLSX 49 kb)
Additional file 4: Table S3. List of GLAM2Scan [24] results for scan of the *Clostridium difficile* R20291 genome for *cd*PadR1 motif (Fig. 5a) for promoters upstream of genes predicted to be involved in transport/efflux and sporulation. The .xlsx file includes the locus ID for the gene(s) nearest the motif (column A); the gene annotation (column B); the genomic placement (start, column C and end, column D); indication of whether the motif is on the leading (+) or lagging (-) strand (column E); the motif sequence generated by GLAM2Scan (column F); and the score generated by GLAM2Scan that indicates how well the sequence fits the *cd*PadR1 motif (column G). (XLSX 39 kb)
Additional file 5: Figure S3. EMSA of *cd*PadR1 binding the 21 bp fragment (Pr68, Additional file 4: Table S1) that contains the inverted repeats TACT/AGTA with 11 nucleotides in between from within its own promoter. Protein-free controls are indicated with a minus sign (-). 21 bp P_*cdpadR1*_ DNA (0.25 μM) was used in a reaction with increasing concentrations of *cd*PadR1 (2.5, 5.0, and 10.0 μM). (PPTX 3693 kb)
Additional file 6: Table S1. Full list of oligonucleotides that were annealed and used in EMSA studies with *cd*PadR1. The .xlsx file includes the arbitrary number assigned to each oligonucleotide (column A); the genomic placement of the minimum nucleotide (column B); length in base pairs (bp, column C); indication of binding (+) or no binding detected (-) (column D); locus tag associated with the gene downstream of the oligonucleotide (column E); the annotated gene downstream of the oligonucleotide (column F); and the oligonucleotide sequence 5′ to 3′ (column G). (XLSX 50 kb)

## Abbreviations

A: Alanine
Å: Ångström
*bc*: *Bacillus cereus*
*bc*PadR1: *Bacillus cereus* locus ID BC4206
*bc*PadR2: *Bacillus cereus* locus ID BCE3449
bp: Base pairs
C: Celsius
CCD: Charged coupled device
*cd*: *Clostridium difficile*
*cd*P_*1590*_: Promoter for locus ID CDR20291_1590
*cd*P_*1882*_: Promoter for locus ID CDR20291_1882
*cd*P_*2322*_: Promoter for locus ID CDR20291_2322
*cd*PadR1: Locus ID CDR20291_0991
*ct*: *Clostridium thermocellum*
C-terminal: Carboxy-terminal
DNA: Deoxyribonucleic acid
dsDNA: Double stranded deoxyribonucleic acid
DTT: Dithiothreitol
EMSA: Electrophoretic mobility shift assay
gDNA: Genomic deoxyribonucleic acid
GLAM2: Gapped local alignment of motifs version 2
GTP: Guanosine triphosphate
H33342: Hoeschst 33342
HEPES: 4-(2-hydroxyethyl)-1-piperazineethanesulfonic acid
HTH: Helix-turn-helix
ID: Identification
I-I: Indole-indole
I-P: Indole-phenol
KCl: Potassium chloride
L: Liter
LmrR: Lactococcal multidrug resistant transcription regulator
M: Molar
MALS: Multi-angle light scattering
MCSG: Midwest Center for Structural Genomics
MEME: Multiple Em for Motif Elicitation
MgCL_2_: Magnesium chloride
mM: Millimolar
MW: Molecular weight
N: Any nucleotide
NaCl: Sodium chloride
NaH_2_PO_4_: Sodium phosphate
NaOH: Sodium hydroxide
*norV*: Nitric oxide reductase gene
N-terminal: Amino-terminal
*obj*: Gene encoding Spo0B
ORF: Open reading frame
PadR: Phenolic acid decarboxylase regulator
PadR-s1: Subfamily of the PadR protein family
PadR-s2: Subfamily of the PadR protein family
P_*cdpadR1*_: Promoter of *cdpadR1* (locus ID CDR20291_0991)
PDB: Protein Data Bank
P_*norV*_: Promoter of *norV* gene
P_*obg*_: Promoter of *obg* gene
P-P: Phenol-phenol
Pr: Promoter
qRT-PCR: quantitative reverse transcriptase polymerase chain reaction
RMSD: root mean square deviation
Spo0B: sporulation initiation phosphotrasferase B
ssDNA: single stranded deoxyribonucleic acid
TB: tris-borate
W: tryptophan
wHTH: winged helix-turn-helix
WT: wild-type
μL: microliter
μM: micromolar
