# Supplementary figures and images for: Crystal structure and DNA binding activity of a PadR family transcription regulator from hypervirulent Clostridium difficile R20291
Source: BMC Microbiol. 2016 Oct 4;16:231. doi: 10.1186/s12866-016-0850-0 (PMC5050560; doi:10.1186/s12866-016-0850-0)

## Slide 1
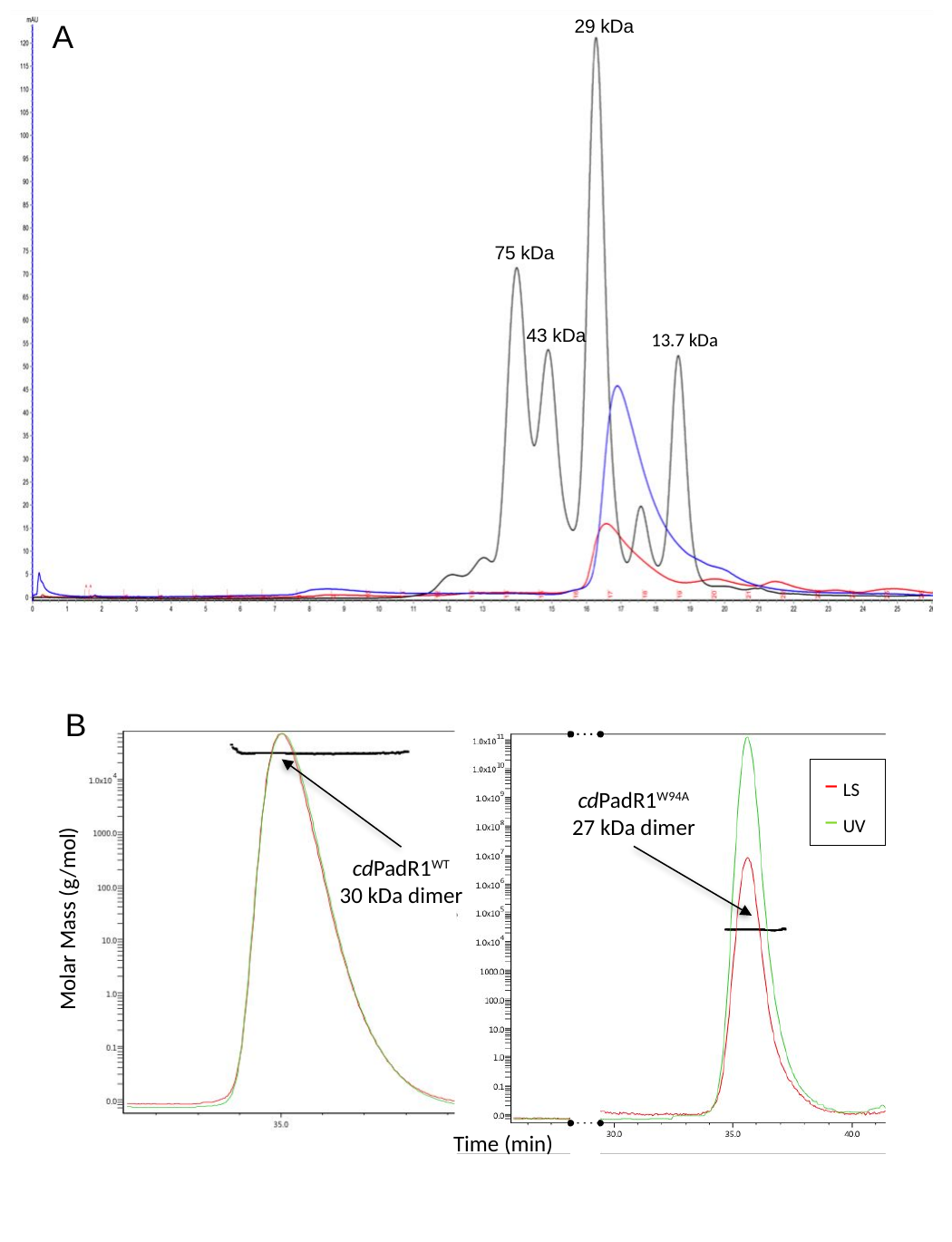

29 kDa
A
75 kDa
43 kDa
13.7 kDa
B
− LS
− UV
cdPadR1W94A
27 kDa dimer
cdPadR1WT
30 kDa dimer
Molar Mass (g/mol)
Time (min)

Supplement: Additional file 1: Figure S1. — (A) Chromatogram from size exclusion chromatography (SEC) run performed on a Superdex 200 Increase 10/300 GL column connected to an ÄKTA Pure 25 (GE Healthcare). The black line represents the calibration standard mix with molecular weights of standards labeled. The blue and red lines indicate the elution profile for cdPadR1WT and cdPadR1W94A, respectively (13.5 kDa monomer size for both). (B) Molar mass versus elution time of cdPadR1WT and cdPadR1W94A from SEC (as described) coupled with multi-angle light scattering (MALS) detection. Red lines indicate MALS signal (LS) and green lines indicate UV detection. cdPadR1WT and cdPadR1W94A both dimers with molecular weights (MW) of approximately 30 and 27 kDa, respectively (monomeric cdPadR1 MW is 13.5 kDa). (PPTX 222 kb) [file 12866_2016_850_MOESM1_ESM.pptx]

## Slide 1
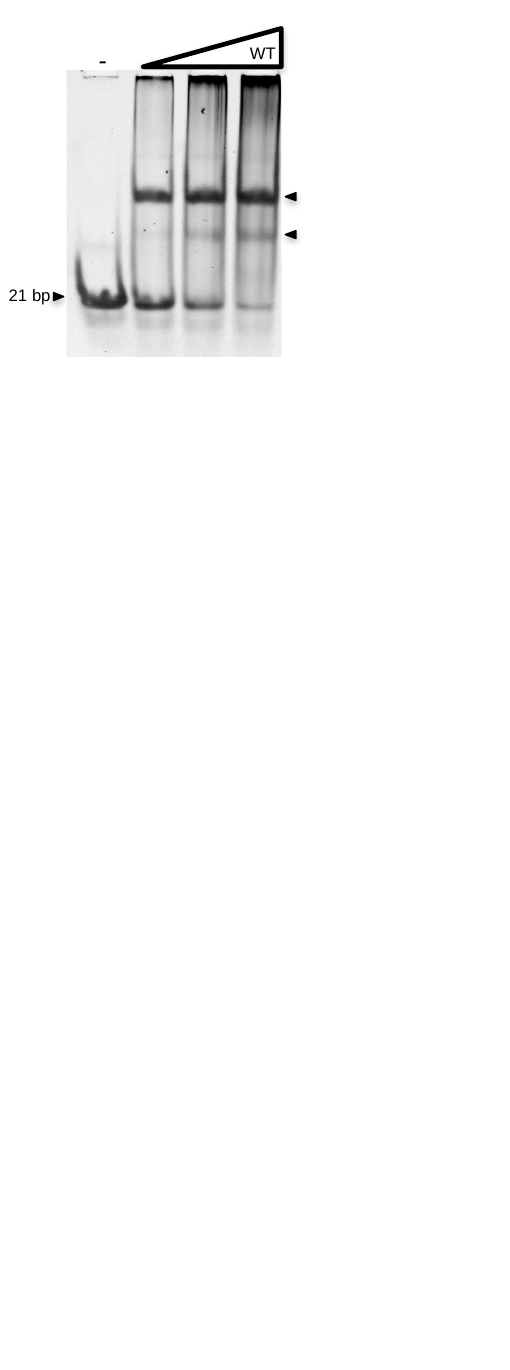

WT
-
21 bp

Supplement: Additional file 5: Figure S3. — EMSA of cdPadR1 binding the 21 bp fragment (Pr68, Additional file 4: Table S1) that contains the inverted repeats TACT/AGTA with 11 nucleotides in between from within its own promoter. Protein-free controls are indicated with a minus sign (-). 21 bp PcdpadR1 DNA (0.25 μM) was used in a reaction with increasing concentrations of cdPadR1 (2.5, 5.0, and 10.0 μM). (PPTX 3693 kb) [file 12866_2016_850_MOESM5_ESM.pptx]
