# Supplementary material for: Crystal structure and DNA binding activity of a PadR family transcription regulator from hypervirulent Clostridium difficile R20291
Source: BMC Microbiol. 2016 Oct 4;16:231. doi: 10.1186/s12866-016-0850-0 (PMC5050560; doi:10.1186/s12866-016-0850-0)
Supplement: Additional file 2: Figure S2. — EMSA of cdPadR1 binding different fragments of PcdpadR1, that contain the inverted repeats TACT/AGTA with 4 bp overhang on the 5′ and 3′ end of inverted repeats. Each dsDNA contains a different number of nucleotides between TACT/AGTA (addition of a central alanine). EMSAs were conducted as described for 100 bp and smaller dsDNA fragments in the materials and methods. A complete list of nucleotides tested is available in the table below the EMSA gels. Inverted repeats are underlined. For Pr101, the AT rich region that was mutated is indicated in bold. The - lane contains DNA only and the + lane contains 10-fold cdPadR1 in excess over DNA. (PPTX 256 kb) [file 12866_2016_850_MOESM2_ESM.pptx]

## Slide 1
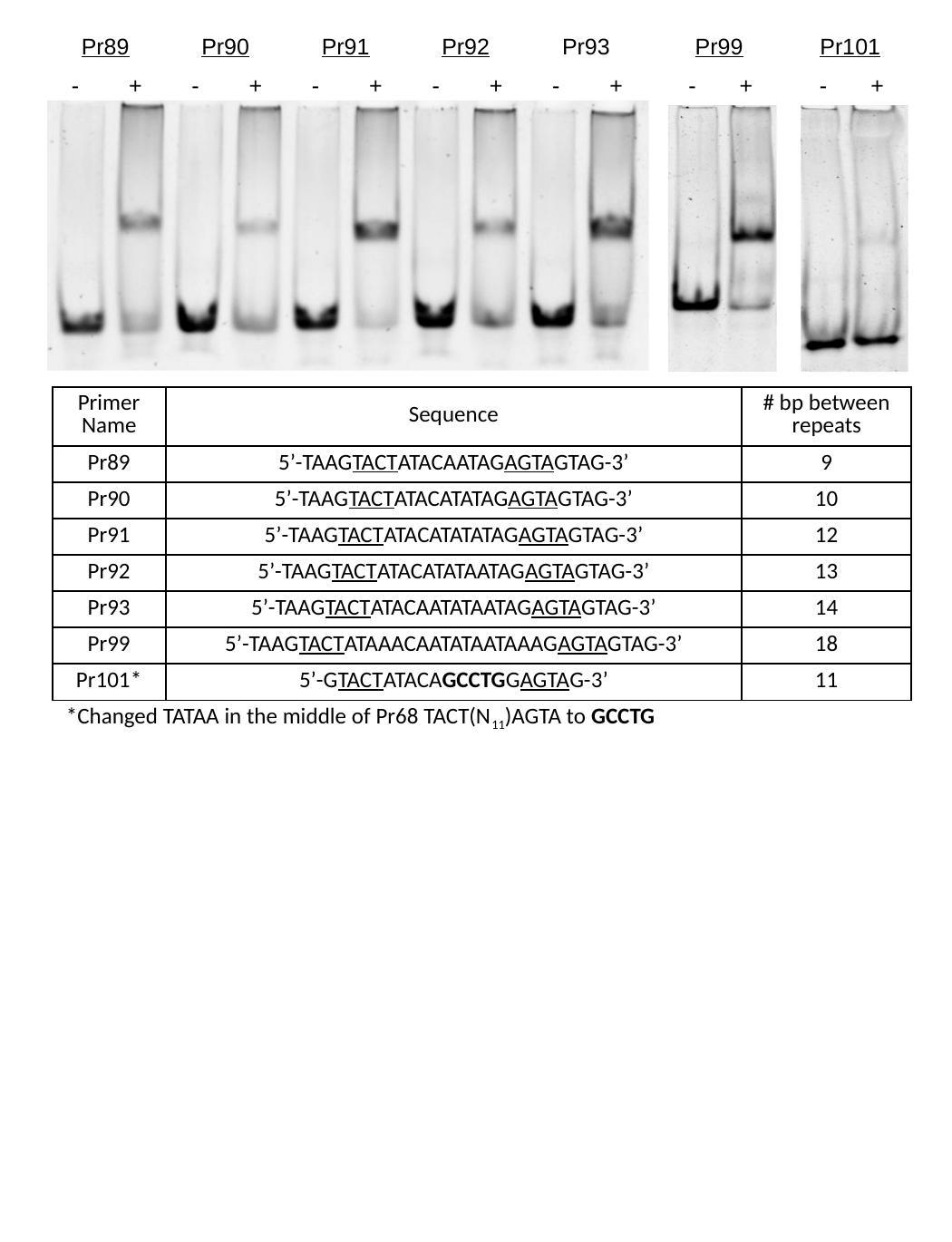

| Pr89 | | Pr90 | | Pr91 | | Pr92 | | Pr93 | |
| --- | --- | --- | --- | --- | --- | --- | --- | --- | --- |
| - | + | - | + | - | + | - | + | - | + |
| Pr99 | |
| --- | --- |
| - | + |
| Pr101 | |
| --- | --- |
| - | + |
| Primer Name | Sequence | # bp between repeats |
| --- | --- | --- |
| Pr89 | 5’-TAAGTACTATACAATAGAGTAGTAG-3’ | 9 |
| Pr90 | 5’-TAAGTACTATACATATAGAGTAGTAG-3’ | 10 |
| Pr91 | 5’-TAAGTACTATACATATATAGAGTAGTAG-3’ | 12 |
| Pr92 | 5’-TAAGTACTATACATATAATAGAGTAGTAG-3’ | 13 |
| Pr93 | 5’-TAAGTACTATACAATATAATAGAGTAGTAG-3’ | 14 |
| Pr99 | 5’-TAAGTACTATAAACAATATAATAAAGAGTAGTAG-3’ | 18 |
| Pr101\* | 5’-GTACTATACAGCCTGGAGTAG-3’ | 11 |
| \*Changed TATAA in the middle of Pr68 TACT(N11)AGTA to GCCTG | | |
